# Ternary mixtures of ionic liquids for better salt solubility, conductivity and cation transference number improvement

**DOI:** 10.1038/srep35587

**Published:** 2016-10-21

**Authors:** E. Karpierz, L. Niedzicki, T. Trzeciak, M. Zawadzki, M. Dranka, J. Zachara, G. Z. Żukowska, A. Bitner-Michalska, W. Wieczorek

**Affiliations:** 1 Warsaw University of Technology, Faculty of Chemistry, Noakowskiego 3, 00-664 Warsaw, Poland

## Abstract

We hereby present the new class of ionic liquid systems in which lithium salt is introduced into the solution as a lithium cation−glyme solvate. This modification leads to the reorganisation of solution structure, which entails release of free mobile lithium cation solvate and hence leads to the significant enhancement of ionic conductivity and lithium cation transference numbers. This new approach in composing electrolytes also enables even three-fold increase of salt concentration in ionic liquids.

Nowadays, lithium−ion batteries (LIBs) are one of the most common energy storage systems − due to their low weight, very high energy density and low self-discharge. The most challenging demands for them are formulated mostly by portable electronics industry and rapid progress therein. However, improvements are still necessary, because currently available storage capacities are still insufficient^1^,^2^,^3^. Although numerous possible ameliorations have been proposed, the major concern for safety, mostly due to use of the volatile and flammable organic solvents used in the commercial electrolytes, is still not adequately solved in the commercial production^1^,^4^.

The ionic liquids (ILs), molten salts with melting point below 100 °C^5^, can be helpful in replacing these dangerous components and improving the safety of LIBs, thanks to their unique physicochemical properties. High ionic conductivity and wide electrochemical window creates the hope of ILs being an excellent solvent for electrolytes used in electrochemical devices^6^,^7^,^8^. However, some limitations of ILs still make the implementation of these solutions in high-power application impossible. The most significant disadvantage is their price originating from the fact that most of them are still synthesized only in the small scale. In comparison to the commonly used organic solvents ILs have higher melting points (sometimes even higher than room temperature) which is connected with the length of alkyl chain substituent in anion/cation. Also their viscosity is higher, what results in lower diffusion coefficients^9^ and lower transference number of lithium cation^10^. The ionic conductivity for higher concentrations of lithium salt in ILs is lower and the compatibility with electrodes is worse^11^. Thus, in general, ILs does not work good as lithium conducing electrolyte in LIBs. Our previous studies based on TDI anions^8^ were attempts motivated by the hope of elimination or reduction these disadvantages. We decided to create a new family of ionic liquids of composition XMIm^+^TDI^−^ with cation selected from imidazolium derivatives family: XMIm^+^ stands for 1-alkyl-3-methylimidazolium cation, where X stands for alkyl, E for ethyl, P for propyl, B for butyl group and TDI^−^ anion stands for 4,5-dicyano-2-(trifluoromethane)imidazole anion. However, attempts to dissolve LiTDI salt in a new imidazolium ionic liqiud with common anion were only partially succesful because of low miscibility, its instability and finally low transference number (*T*_Li+_ = 0.04)^8^. It was noticed that the conductivity and transference numbers visibly were decreased with increasing viscosity. These changes are associated with creating a 3 dimensional net structure. We have found XMIm^+^TDI^−^-LiTDI system to form, at higher salt concentration, a crystalline complex where lithium cations and imidazolium anions create chain-shaped [Li(TDI)_2_]_n_^n−^ polyanions which are surrounded by XMIm^+^ cations. Such a structure is a very good conductor of imidazolium anions, but not of lithium cations. The desirable charge carriers are immobilized resulting in low value of conductivity and *T*_Li+_ and the high viscosity. Therefore, increasing the salt content in such system leads only to higher aggregation, where almost all lithium cations are permanently immobilized.

On the other hand, we have investigated the systems containing LiTDI salt and glymes^12^,^13^. The papers by Jankowski *et al*. show that use the glyme in the appropriate low ratio can lead to the increase of such parameters as: ionic conductivity and lithium cation transference number. These facts are explained by disproportionation of the system with forming a stable solvated cations Li(glyme)_n_^+^, and aggregated lithium polyanions. The fraction of free mobile cations, which are solvent-coordinated small ions are responsible for fast and effective transfer of charge. Aggregated polyanions are immobilized and are responsible for observed higher transference numbers. In particular for (already solid) tetraglyme based electrolyte with Li:O ratio equal to 1:1.67 an exceptionally high value of lithium transference number of *T*_Li+_ = 0.82 was observed at 30 °C for the complex with tetraglyme^12^. However, such approach results in crystalline or solid material characterized by high melting temperatures.

Herein, we present a new concept based on a simple, but novel idea, that addition of glyme into BMIm^+^TDI^−^-LiTDI system should enable solving much more salt than in the system without glyme. Such ternary mixtures containing lithium salt, glyme and ionic liquid can be consider from structural point of view as an aggregated [Li(glyme)]^+^ [Li(TDI)_2_]_n_^n−^ system dissolved in ionic liquid like BMIm^+^TDI^−^ in which both structures: that of glyme solvate lithium cations and that of lithium aggregated polyanions are preserved. In this paper we present results confirming this idea.

## Results and Discussion

The scope of our investigations was solution of three components: salt – LiTDI, ionic liquid – BMImTDI and glyme – triglyme (3G) or tetraglyme (4G). We have found that the way of mixing components play an important role in further properties of the mixture. We used two methods of preparing the final solution. In both methods the system was prepared in two-steps procedure.

In the method 1 - lithium salt LiTDI was dissolved in the tri- or tetraglyme and after at least six hours of stirring the BMImTDI ionic liquid was added.

In the method 2 - the salt was dissolved in ionic liquid and after the same time of stirring glyme was added.

Then the conductivity and viscosity of the mixture were measured and analysed. Selected results for tetraglyme are shown in the Table 1a,b. Comparing section 1a & 1b one can see that for method 1 one get higher conductivity while viscosity remains almost the same for both methods. This is especially noticeable for higher temperatures.

General conclusion is that the addition of glyme improves conductivity, but the order of adding components visibly affects the results. They can be understood taking into account that at higher temperatures ion−ion interactions are more important^14^. Therefore reduction of aggregates formation via addition of already created lithium−glyme solvates results in higher conductivity.

For further investigations the method 1 of mixing components was always used.

As it was mentioned the investigated systems consisted of three components: salt – LiTDI, ionic liquid – BMImTDI and tri- or tetraglyme. Ratios between those components were chosen in the following way:

1. The salt and glyme were mixed in molar ratio 1:*y*, where *y* = 2 or 5.6 in case of triglyme and *y* = 1, 2 or 4.5 in case of tetraglyme.
2. The ionic liquid was added to the glyme−salt solutions creating the system: *x*[LiTDI **·** *y*(glyme)]: (1−*x*)BMImTDI.

**Figure 1:**
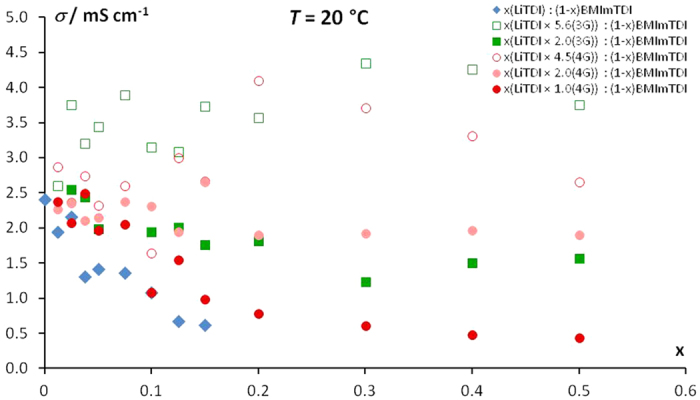
Dependence of ionic conductivity at 20 °C on LiTDI content in BMImTDI with the addition of triglyme with molar ratio of 1:2 and 1:5.6 and tetraglyme with molar ratio of 1:1, 1:2 and 1:4.5; the results of two-component systems were added for comparison^8^.

During the preparation of electrolytes it was noticed that the addition of glyme significantly improves the ability of salt dissolution by the ionic liquid and it allowed to obtain very high miscibility of salt in IL. This is a reason why at the beginning we have done the conductivity measurements for the solutions with salt content up to *x* = 0.5 (see Fig. 1). It was possible because of using glyme, while in pure BMImTDI the highest obtainable molar fraction of LiTDI was *x* = 0.15. In order to properly compare obtained results for ternary and binary systems, only 4 solutions were chosen for further investigation: *x* = 0.025, 0.050, 0.100 and 0.150. Only for this range of compositions it was possible to compare the results of ternary systems with relevant ones of binary systems.

The solution of LiTDI in glyme was prepared in molar ratio of 1:2 and 1:5.6 in case of 3G and 1:1, 1:2 and 1:4.5 in case of 4G. In tetraglyme it was possible to obtain higher salt concentration than in triglyme. In 4G the maximum solubility of LiTDI is slightly higher than molar ratio 1:1, while for 3G the obtained maximum of solubility of LiTDI is 1:2.

The ionic conductivity plot at 20 °C for the ternary systems compared with that obtained for the binary one (see Fig. 1) reveals that addition of solvates even with a small amount of oligomers increases the ionic conductivity for both glymes. But the extent of this change depends on a glyme used. In case of triglyme higher addition means better conductivity, even four-fold for the 1:2 salt−3G ratio and up to seven-fold for highly diluted salt−glyme solution (1:5.6 ratio). Whereas, in the case of tetraglyme ionic conductivity in all solutions increases about 2–3 times. Observed improvement is less visible comparing to that in triglyme system. The raise of conductivity is particularly evident for samples with more than 0.15 fraction of lithium salts and higher glyme to salt ratios. This is due to changes in viscosity as shown in Supplementary Table 1. In this salt concentration range viscosity decreases for samples with higher glyme to salt ratios whereas the opposite trend is observed for electrolytes with lower glyme to salt ratios.

**Figure 2:**
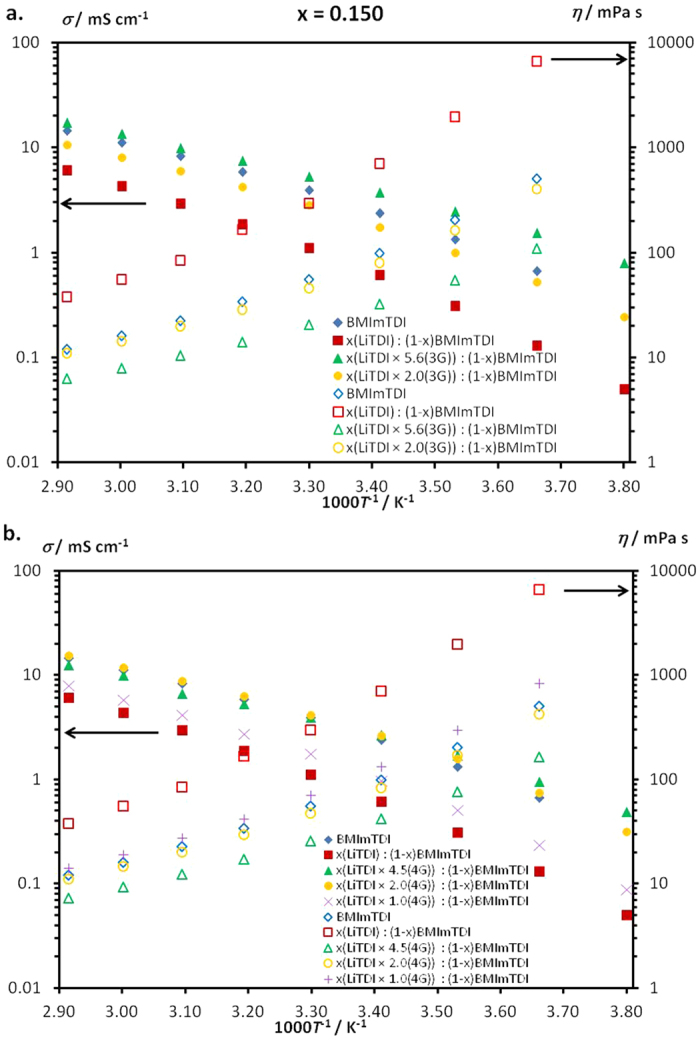
Inverse temperature dependence (1000*T*^*−*1^) of ionic conductivity and viscosity for LiTDI−BMImTDI−glyme systems for mole fraction of salt *x* = 0.150 containing (**a**). triglyme (3G), (**b**). tetraglyme (4G). The results of two-component systems were added for comparison^8^. Temperature 20 °C corresponds to the value 3.41 on the x axis and temperature 70 °C corresponds to the value 2.91 on the x axis.

In Fig. 2 the temperature dependence of ionic conductivity for addition of both glymes solvates is shown for the mostly concentrated systems, i.e. for the mole fraction *x* = 0.150. It is noticeable that adding the salt to the pure ionic liquid causes a significant decrease in ionic conductivity. On the other hand, after salt−glyme solvates addition the opposite effect is observed: comparable or higher conductivity with respect to the pure IL. Using 3G in 1:2 molar ratio allows to improve the ionic conductivity values up to five times, while using 1:5.6 molar ratio brings even sixteen-fold increase for the electrolyte at the highest measured temperature of 70 °C. At the temperature of 20 °C the values of * σ* increases from 0.61 mS·cm^−1^ for two-components system up to 1.75 m·cm^−1^ for lower glyme content solution (1:2) and 3.73 mS·cm^−1^ for the higher one (1:5.6). It makes possible about six-fold * σ* rise. These measurements are supported by the viscosity plots (Fig. 2). As a general rule, ionic conductivity is inversely proportional to viscosity. All measured systems behave according to this rule. That confirms the conclusions from the *σ* measurements analysis that addition of oligomers results in increase of conductivity and decrease of viscosity. These observations are closely related to the system dissociation and creation of free ions. These temperature dependences of ionic conductivity as well as viscosity can be observed for all measured ternary systems (for *x* = 0.025, 0.050, 0.100, 0.150). The figures for all electrolytes are presented in a Supplementary information to this paper (Supplementary Figure 1–3). From all these experiments it can be concluded that the increase in conductivity for ternary system in comparison to the binary one is higher the higher is the salt concentration in the electrolyte. The effect is rather small for 0.025 sample but substantial increase is observed for samples with higher salt concentration. Also at lower temperature the effect of viscosity seems to be predominant (higher conductivities for samples with higher glyme to salt ratio; higher conductivities for triglyme compared with tetraglyme electrolytes) but at elevated temperatures the ionic interactions seem to be more important leading to smaller differences in conductivity for concentrated electrolytes compared with more diluted systems. Similar observation we have made for other ternary systems with different ILs, 3G or 4G and lithium, sodium or magnesium salts^15^.

**Figure 3:**
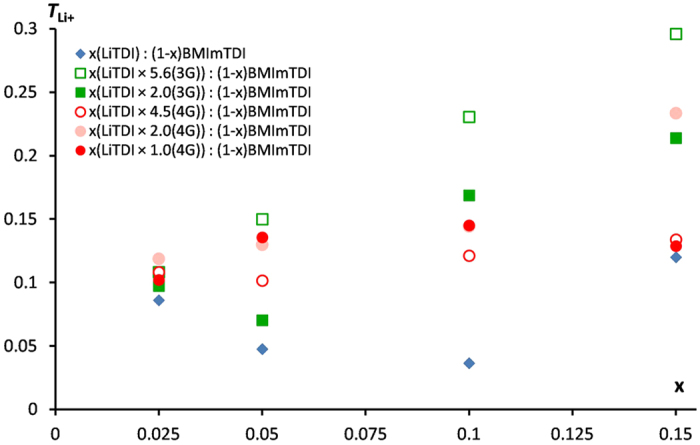
Dependence of lithium cation transference number dependence on LiTDI content in LiTDI−BMImTDI−glyme mixtures at room temperature for (**a**). Triglyme, (**b**). Tetraglyme. All values were averaged over at least three samples.

Lithium cation transference number (*T*_Li+_) was measured for all the investigated mixtures. The results are shown in Fig. 3. Ternary electrolytes exhibit significantly better values of *T*_Li+_ with respect to those of salt−IL mixtures. For the lower concentrations of salt the differences are rather slight, but for all measured systems the increase of *T*_Li+_ is evident. For higher molar fraction of salt the highest increase is observed for *x* = 0.1, where the increase is almost five-fold. The transference number measurements are the best way to deduce what is the kind of ions responsible for the charge transport. The total conductivity increases, but *T*_Li+_ measurements show that mainly lithium cations are responsible for this phenomenon. *T*_Li+_ value increase can be explained by the release of lithium cation solvates from the polyanionic aggregation. Thus, the asymmetry in the presence of positive and negative charge carriers appears. This can be related to the higher degree of anion aggregation.

**Figure 4:**
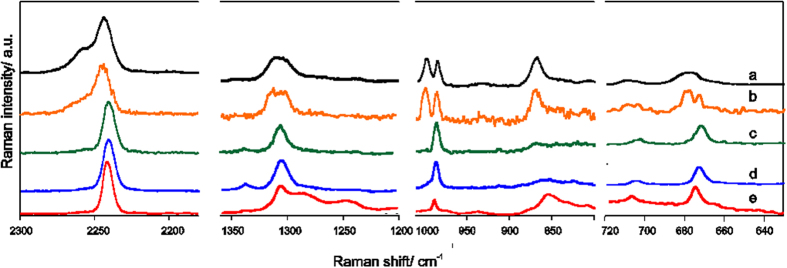
Raman spectra of samples: (**a**) molten LiTDI-4G. (**b**) 0.1[LiTDI**·**4.5(4G)]:0.9BMImTDI (subtraction spectrum), (**c**) 0.1[LiTDI**·**2.0(4G)]:0.9BMImTDI (subtraction spectrum), (**d**) 0.1[LiTDI**·**1.0(4G)]:0.9BMImTDI (subtraction spectrum), (**e**) BMImTDI.

It was shown in our former work^8^ that anions in the pure ionic liquid can be considered as “spectroscopically free”, while in BMImTDI-LiTDI crystalline complex form a ladder-like structure, built from [Li(TDI)_2_]_n_^n−^ polyanions. As a result, characteristic bands of anions in Raman spectrum of the BMImTDI-LiTDI, i.e. *ν*_CN_ (2261, 2248 cm^−1^), *ν*_CN Im_ (1318, 1307 cm^−1^) and *δ*_NCN_ (996 cm^−1^) are split and their maxima are shifted towards higher values as compared with spectrum of BMImTDI (see Supplementary Figure 4b). Further changes were observed in spectra of ternary electrolytes. Figure 4 presents a comparison of Raman spectra of molten crystals LiTDI-4G, 0.25 M LiTDI-4G solution and subtraction spectra of BMImTDI-LiTDI-4G electrolytes with 1:1, 1:2 and 1:4.5 LiTDI:4G ratios. It was found previously, that structure of LiTDI-4G complex consists from polyanions and dications, the latter formed by two Li cations wrapped by two 4G chains. After melting the polyanions disappear and the anions exist in form of dimers or chains (compare Supplementary Figure 5). In the studied ternary systems the salt molar fraction is 0.1 and the spectral pattern is dominated by the ionic liquid. Therefore, spectrum of pure ionic liquid were mathematically subtracted from the electrolyte’s spectra, in order to unshield the spectral feature of LiTDI complex. As shown, an addition of glyme modifies the electrolyte’s structure but doesn’t simply lead to the formation of free anions or ionic pairs. Instead, at the highest (1:1) LiTDI:4G ratio the spectral pattern resembles the spectrum of molten LiTDI-4G complex, while in systems with lower LiTDI:4G ratio the position of the bands points on the formation of different types of solvates, representing ionic pairs and free ions. For more Raman spectra see Supplementary Figure 4a–d. It can be concluded, that the presence both of fully solvated cations or dications and aggregated lithium polyanions are responsible for an increase of lithium transference numbers observed for the samples with higher salt content.

**Figure 5:**
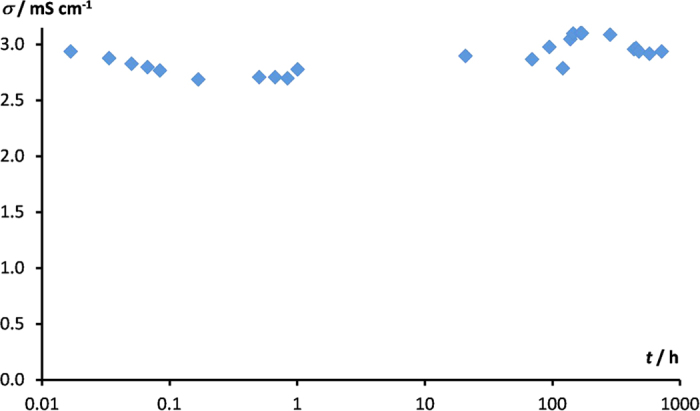
Time dependence of ionic conductivity for 0.15[LiTDI·4.5(4G)]:0.85BMImTDI system at room temperature.

It can be considered whether such systems are metastable. To prove that it is not the case, conductivity measurements over the wide range of temperatures from −10 °C to 70 °C were conducted. In order to test the stability of the resulting lithium salt−glyme solvates, the time dependency of conductivity was investigated. If the solvate was not stable and rearranged spontaneously, the association degree with ionic liquid would increase resulting in ionic conductivity decrease. As shown in Fig. 5, the conductivity does not change even after one month. Thus, it can be concluded, that the solvate is stable during this period of time.

**Figure 6:**
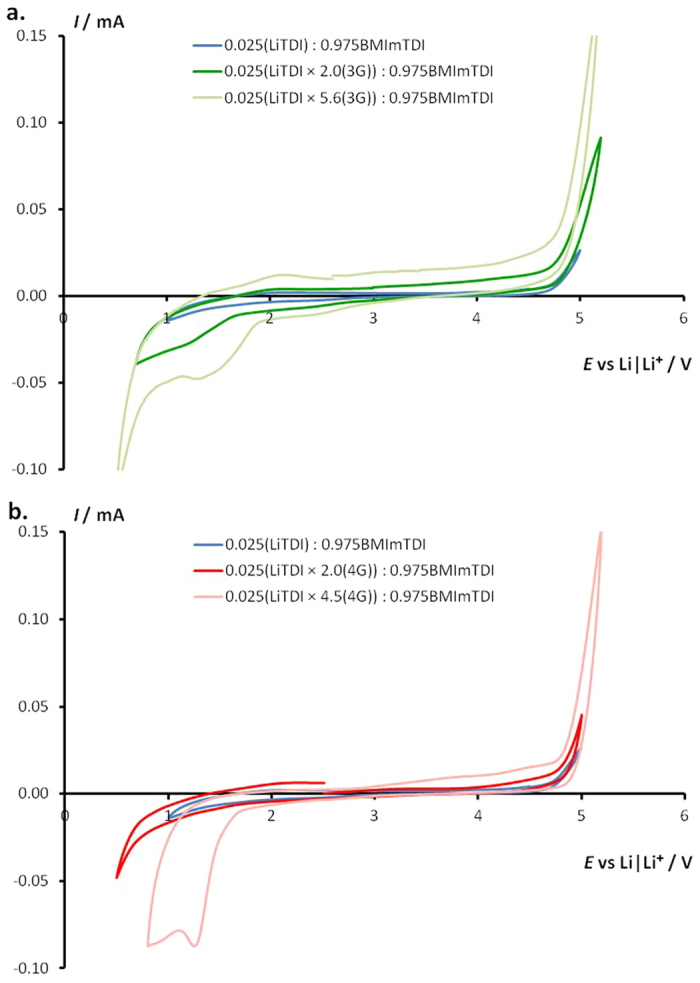
Cyclic voltammoram (CV) of ternary systems 0.025[LiTDI**·***y*(glyme)]:0.975BMImTDI at room temperature containing (**a**). triglyme (*y* = 2.0 or 5.6), (**b**). tetraglyme (*y* = 2.0 or 4.5). Metallic lithium acts both counter and reference electrodes. The scan rate was 1 mVs^−1^.

For the very first time we have shown that the exact stoichiometric amount of oligomer is essential for electrochemical stability of such ternary system. The electrochemical stability experiment was done for the binary system of 0.025LiTDI:0.975BMImTDI and for different addition of both glymes. In Fig. 6a,b these results are shown for triglyme (Fig. 6a) and tetraglyme systems (Fig. 6b). In each case the results are shown for two glyme to salt ratios and compared with the cyclic voltammetry (CV) results obtained for binary system. The upper limits of stability of all of these electrolytes reach 4.6 V *vs.* Li and it is the stability limit of the LiTDI salt anion^16^. For the lower glyme concentration the stability of the system is comparable to the binary system and its lower limit reach 1.0 V *vs.* Li, while with the increase of oligomer’s amount the reduction peak at higher potentials is observed at 1.7 V *vs.* Li. This peak is caused by the decomposition of redundant glyme unbound with lithium cations. Reducing the amount of unbounded glyme next to the electrodes can be favorable. Properly selected system can improve electrochemical properties through increasing the electrode’s potential^17^. These measurements show not only that these electrolytes’ electrochemical stability is sufficient for modern electrode materials use in LIBs, but also that the amount of glyme content is critical for obtaining the optimal composition of electrolyte.

The first attempts of applying investigated electrolytes with commercial cathode materials were made. The cycling performance of the LiNi_0.5_Mn_1.5_O_4_ cathode material in 0.15[LiTDI**·**2.0(3G)]:0.85BMImTDI is illustrated in Supplementary Figure 6a. The galvanostatic charge–discharge cycling was continued during a week at *C*/20 rate and showed 118.94 mAh g^−1^ initial capacity. Between charge-discharge test impedance spectroscopy has been tested. Supplementary Figure 6b gives a proof that between the cycles electrolyte does not decompose. Our studies show that this electrolyte is fully compatible with cathode material and might be successfully applied in lithium-ion cell as an electrolyte.

## Conclusions

In summary, we have presented a new method for improvement of ionic liquid−lithium salt binary system as an electrolyte for lithium ion cells. Use of glyme enables creation of lithium glyme-coordinated free cation and aggregated polyanion. Such a synergistic effect of imidazolium cations and glyme on lithium electrolyte described in this paper have never been observed before. It can be concluded that ionic liquids like BMIm^+^TDI^−^ are excellent solvent to dissolve aggregated [Li(glyme)]^+^ [Li(TDI)_2_]_n_^n−^ systems preserving structure of glyme solvate lithium cations and aggregated polyanions. The presented results confirm the possibility of obtaining higher conductivity values in comparison to the reference binary systems. By using this method it is also possible to introduce much more salt into the ionic liquid rather than via conventional dissolving. This property is observed for both examined glymes, but for each the trend is different. More detailed measurements were performed for molar fraction of LiTDI *x* ≤ 0.15, as for this range comparison of results with the analogous binary system was possible. The addition of even small amount of oligomers significantly increases conductivity about six-fold on the average. Conductivity measurements were supplemented by the viscosity plots showing the decrease of viscosity compared with binary systems. It was also confirmed that such ternary systems are not metastable and do not decompose after one month. All those ternary systems are also electrochemically stable. Lithium cation transference numbers values increase with the higher Li^+^ molar ratio, inversely to the binary systems. It is explained by the binding of anions in polyanionic aggregates while free glyme-solvated lithium cation are released from these structures. The improvement is observed in all investigated systems. For the 1:5.6 salt:3G ratio system values of *T*_Li+_ reach 0.23 in comparison to the 0.04 for the same binary system. In the paper by Jankowski *et al*.^12^,^13^ it is presented that the *T*_Li+_ value obtained using this method can reach even 0.82.

It can be assumed that such an effect can be observed for other families of ionic liquids as well as other kinds of salts, also more and more investigated lithium, sodium and magnesium salts^15^,^18^,^19^. For each system studied conductivities of ternary electrolytes are higher compared with binary systems. It expands applicability of the other anions, because in view of the glyme usage the cation is more stable and mobile (it has got higher transference number). It also creates the possibility for the ionic liquids where ions’ properties are promising but the salt solubility is very low or even equal zero.

## Methods

Lithium (4,5-dicyano-2-(trifluoromethane)imidazole) (LiTDI) synthesis route^16^ as well as 1-butyl-3-methylimidazolium 4,5-dicyano-2-(trifluoromethyl)imidazolide (BMImTDI) synthesis route^8^ were described previously. Triethylene glycol dimethyl ether (triglyme, 3G) and tetraethylene glycol dimethyl ether (tetraglyme, 4G) were purchased from Sigma Aldrich and used as received. All reagents were stored and samples for measurements were assembled in argon-filled glovebox with moisture level below 1 ppm. Prior to the assembly, the salt and ionic liquid were vacuum dried for 48 hours at 120 °C.

### General procedure of sample preparation

Order of addition of the components in the ternary system is important. In the method bringing better results during the first step LiTDI salt and tri- or tetraglyme were mixed in different molar ratios and stirred for at least 12 hours at room temperature to obtain homogeneous liquids and create lithium−glyme solvate. BMImTDI was added then to the resulting mixture and stirred again for at least 4 hours at room temperature.

In the second method, used only in a very early stage of research, during the first step LiTDI salt and BMImTDI were mixed in different mole fraction and stirred for at least 12 hours at room temperature to obtain homogeneous liquids. Tri- or tetraglyme was added then to the resulting mixture in an appropriate molar ratio and stirred again for at least 4 hours at room temperature.

Ionic conductivity and transference number measurements were carried out on the computer-interfaced VMP3 multichannel potentiostat (Bio-Logic Science Instruments) with frequency response analyser option.

For ionic conductivity measurements electrochemical impedance spectroscopy (EIS) was employed and samples were thermostated in Haake K 75 cryostat-thermostat system with a DC 50 temperature controller where temperature was varied from −10 to 70 °C in 10 °C increments (with a precision of 0.05 °C), allowing an hour for stabilization. Ionic conductivities in electrolytes were measured employing calibrated conductivity cells with cell constant values determined with a 0.1% precision.

Lithium cation transference number (*T*_Li+_) was determined using dc polarization method combined with ac impedance method introduced by Bruce and Vincent^20^ using the following equation:


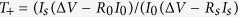


where: Δ*V*−dc polarization voltage applied equal to 20 mV; *R*_0_−initial interfacial layer resistance; *R*_s_ – steady-state interfacial layer resistance; *I*_0_ – initial current; *I*_s_ – steady-state current.

The Li | electrolyte | Li cells were used for transference number experiments. Polypropylene separator was soaked with the electrolyte and sandwiched between two lithium electrodes in symmetrical Swagelok-type cell. EIS used to obtain *R*_0_ and *R*_s_ has been performed with 5 mV amplitude over the 500 kHz–100 mHz frequency range with 10 points per decade. At least three samples have been measured for each electrolyte composition for more consistent data. Detailed description of this method can be found in other papers^21^. Exemplary EIS and dc polarization plots are shown in Supplementary Figure 7 in the supplementary material.

The Raman spectra were collected on a Nicolet Almega Raman dispersive spectrometer. Diode laser with excitation line 780 nm was used. The spectral resolution for all experiments was about 2 cm^–1^. The exposition time was set to 45 s. Temperature-dependent spectra were obtained with the use of a Peltier cooled Linkam stage.

Viscosity measurements were executed using a Physica MCR301 Anton Paar Rheometer with a CP40 cone plate and thermoelectric heat pump base for thermostating. An appropriate amount of electrolyte was thermostated with 0.01 °C precision and measured in 0 to 70 °C temperature range in 10 °C increments with a shear rate varied from 10 to 1000 s^−1^.

Cyclic voltammetry (CV) was used to investigate the electrochemical stability window of systems at room temperature. Sample was sandwiched between metallic lithium (both counter and reference electrodes) and stainless steel (working electrode). It was performed with the 1 mV·s^−1^ scan rate.

Electrochemical characterization was conducted in a custom-made, argon-filled glove box (O_2_ and H_2_O <1.4 ppm) with a Biologic Science Instruments VMP3 multipotentiostat station. Galvanostatic cycling was continued at *C*/20 rate. Sample cells were prepared in a Swagelock-type geometry (coin-cell) cells with lithium foil counter electrode and LiNi_0.5_Mn_1.5_O_4_ cathode material. Glassy fiber separators were immersed in the electrolyte solution and the electrochemical test were performed. Cycling voltage was set in the 2.0–4.2 V range. Before charge-discharge test and after 5 cycles of charge-discharge the Impedance Spectroscopy test were done. Measurements of impedance spectra were recorded at frequencies range from 10 MHz to 100 mHz, collecting 10 points per decade.

## Additional Information

**How to cite this article**: Karpierz, E. *et al*. Ternary mixtures of ionic liquids for better salt solubility, conductivity and cation transference number improvement. *Sci. Rep.* **6**, 35587; doi: 10.1038/srep35587 (2016).

## Supplementary Material

Supplementary Information

## Figures and Tables

**Table 1:** The comparison of a. ionic conductivity (*σ*) and b. viscosity (*η*) for the ternary system obtained in two different possible ways: 1. dissolving salt in the glyme, and as the second step adding IL, 2. dissolving salt in the IL, and as a second step adding glyme.

*y*	method 1 20 °C	method 2 20 °C	method 1 70 °C	method 2 70 °C
***σ*****/mS·cm**^**−1**^				
**a.**				
0	2.15	2.15	13.33	13.33
0.5	1.93	2.21	32.62	21.44
1.0	1.97	1.96	15.62	10.95
2.0	2.10	2.09	42.63	17.97
***η*****/mPa s**				
**b.**				
0	120.91	120.91	13.29	13.29
0.5	85.29	88.78	10.29	11.06
1.0	88.13	81.20	11.01	10.43
2.0	80.46	92.17	10.40	11.60

It was measured for 0.025[LiTDI**·**y(4G)]:0.975BMImTDI system, where *y* = 0, 0.5, 1.0, 2.0 at 20 °C and 70 °C.
