# Supplementary material for: Ternary mixtures of ionic liquids for better salt solubility, conductivity and cation transference number improvement
Source: Sci Rep. 2016 Oct 21;6:35587. doi: 10.1038/srep35587 (PMC5073297; doi:10.1038/srep35587)
Supplement: Supplementary Information [file srep35587-s1.doc]

**Supplementary Information**

**Ternary mixtures of ionic liquids for better salt solubility, conductivity and cation transference number improvement**

E. Karpierz^1*^, L. Niedzicki^1^, T. Trzeciak^1^, M. Zawadzki^1^, M. Dranka^1^, J. Zachara^1^, G. Z. Żukowska^1^, A. Bitner-Michalska^1^, W. Wieczorek^1^

^1^ Warsaw University of Technology, Faculty of Chemistry, Noakowskiego 3, Warsaw, 00-664, Poland

**Figures**

a.

b.

Supplementary Figure 1. Inverse temperature dependence (1000*T^−^*^1^) of ionic conductivity and viscosity for LiTDI−BMImTDI−glyme systems for mole fraction of salt *x* = 0.025 containing a. triglyme (3G), b. tetraglyme (4G). The results of two-component systems were added for comparison [8].

a.

b.

Supplementary Figure 2. Inverse temperature dependence (1000*T^−^*^1^) of ionic conductivity and viscosity for LiTDI−BMImTDI−glyme systems for mole fraction of salt *x* = 0.050 containing a. triglyme (3G), b. tetraglyme (4G). The results of two-component systems were added for comparison [8].

a.

b.

Supplementary Figure 3. Inverse temperature dependence (1000*T^−^*^1^) of ionic conductivity and viscosity for LiTDI−BMImTDI−glyme systems for mole fraction of salt *x* = 0.100 containing a. triglyme (3G), b. tetraglyme (4G). The results of two-component systems were added for comparison [8].

a.
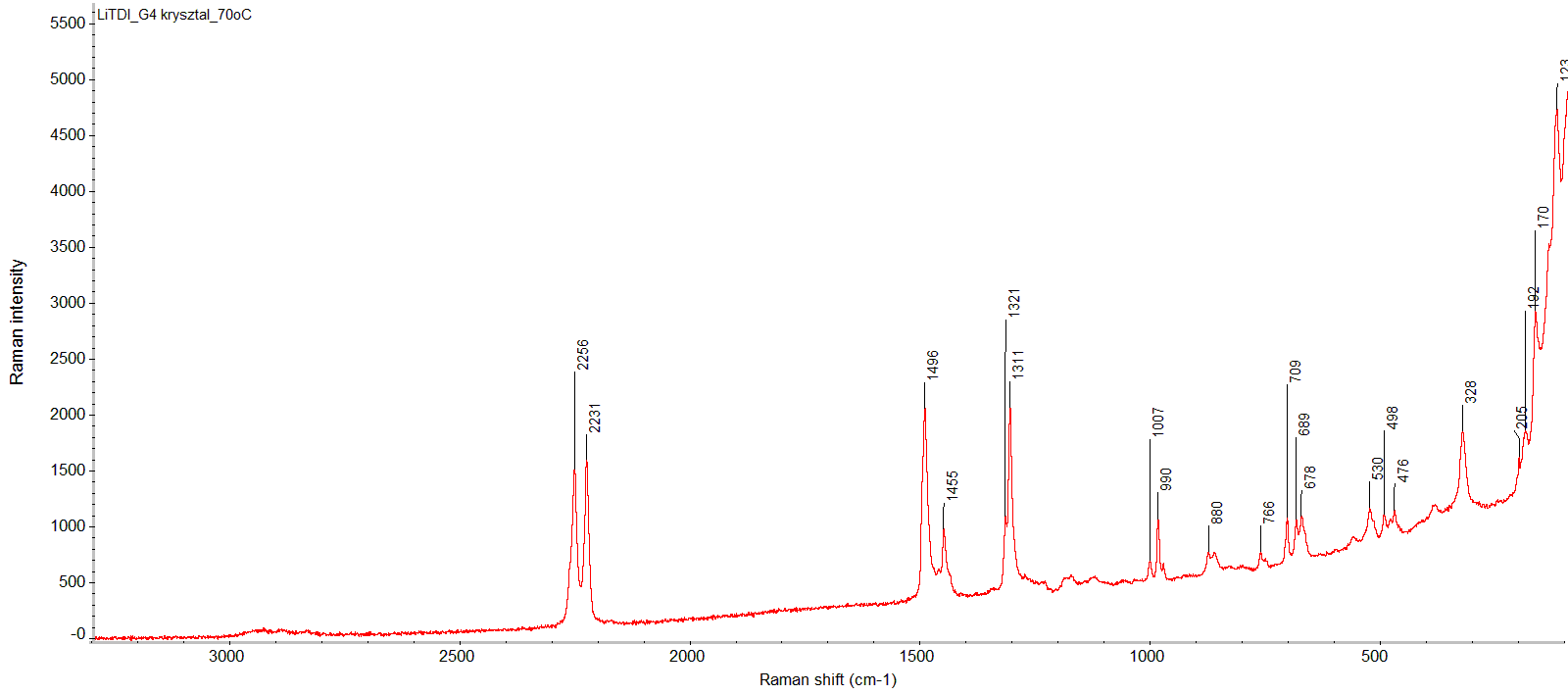


b.
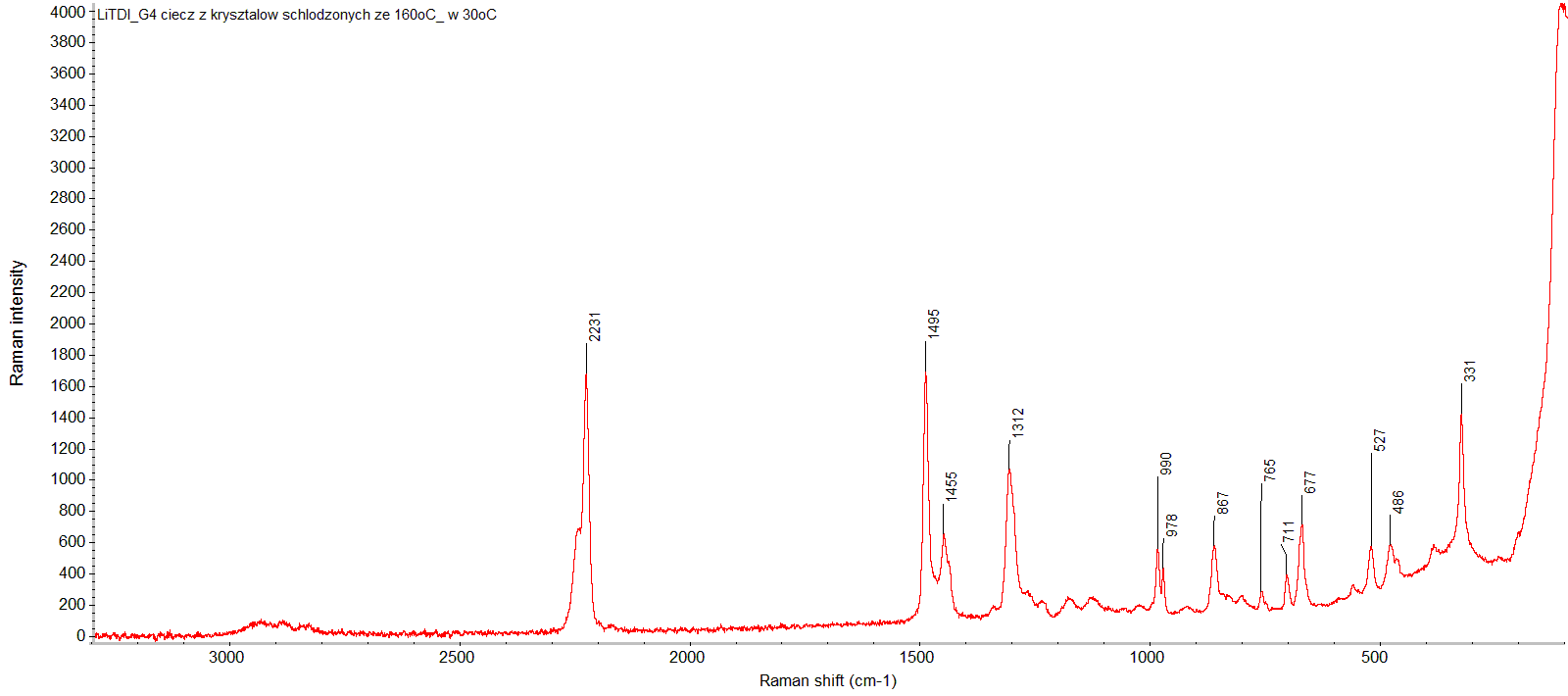


c.
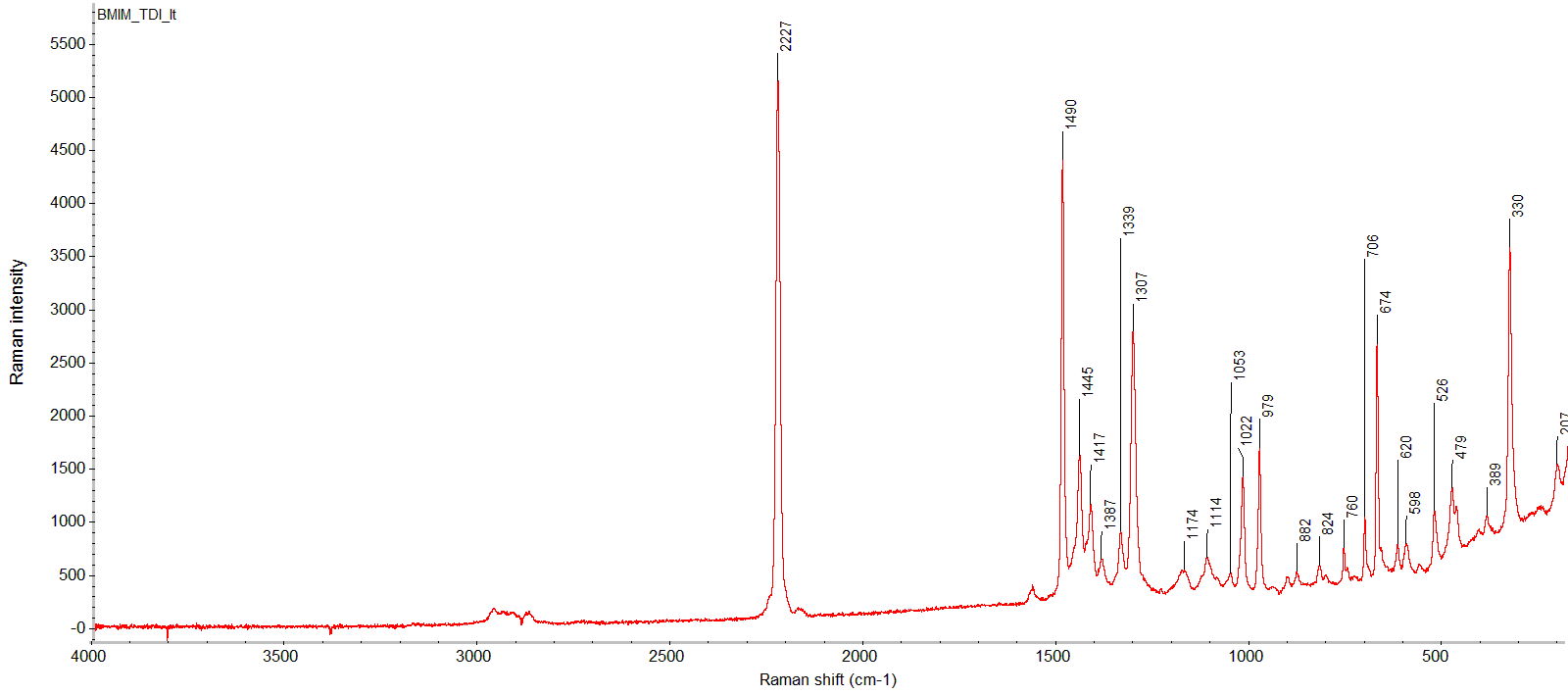


d.
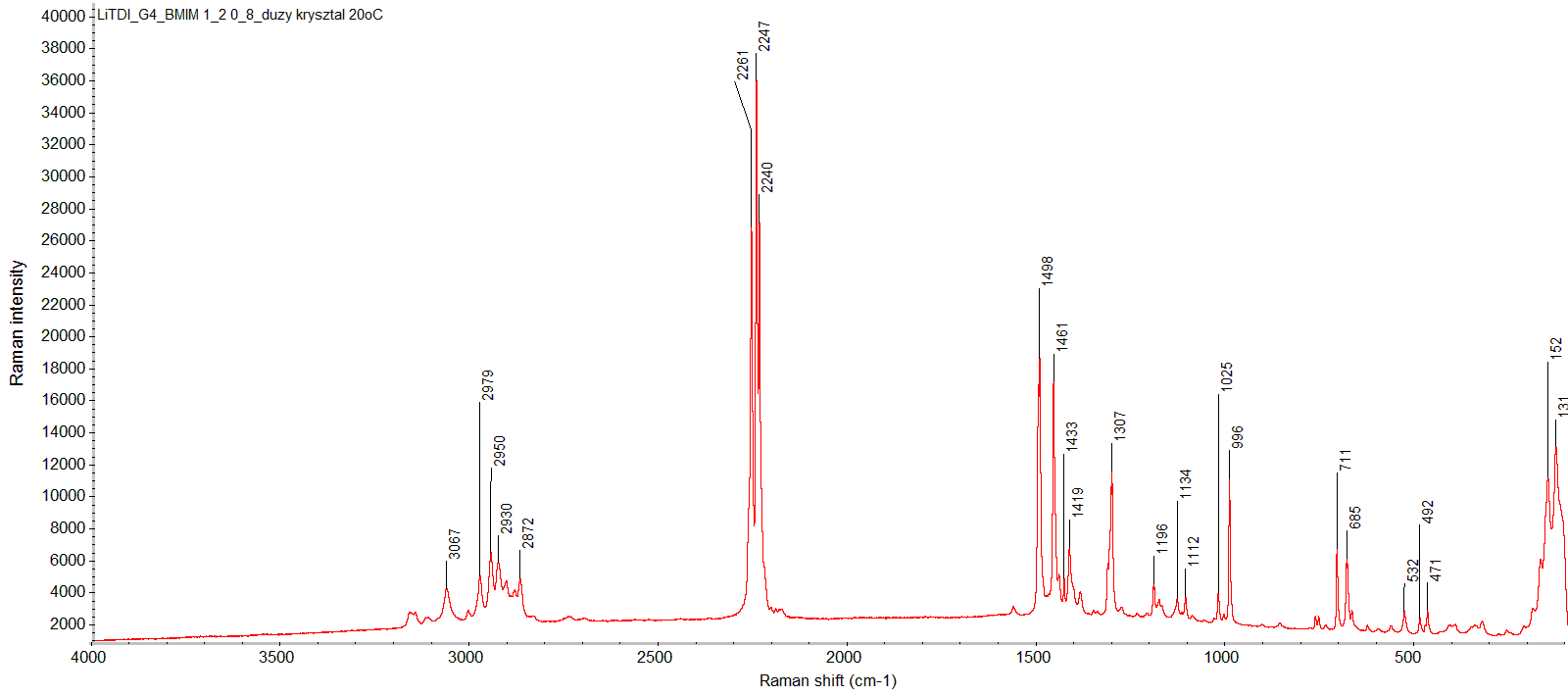


Supplementary Figure 4. Raman spectrum of a. crystalline solvate of [Li_2_(4G)_2_^2+^][Li_4_(TDI)_6_^2−^], b. [Li_2_(4G)_2_^2+^][Li_4_(TDI)_6_^2−^] after melting and cooling to 30°C, c. BMImTDI, d. BMImTDI-LiTDI crystal.

Supplementary Figure 5. Raman spectra of LiTDI-4G complex recorded at various temperatures.

a.

b.

Supplementary Figure 6. a. Discharge capacities during cycling of LiNi_0.5_Mn_1.5_O_4_ | electrolyte | Li half-cell at *C*/20 rate, where electrolyte is 0.15[LiTDI**·**2.0(3G)]:0.85BMImTDI. b. Impedance spectroscopy of the same half-cell at *C*/20 rate.

Supplementary Figure 7. Chronoamperometry of the Li | electrolyte | Li cell at a potential step of 5 mV, where the electrolyte was 0.15[LiTDI**·**4.5(4G)]:0.85BMImTDI. Inset: the ac impedance spectra of the same cell before polarization and after the steady-state current.

**Tables**

Supplementary Table 1. The comparison of viscosity (*η*) for the ternary system obtained via 1. method (dissolving salt in the glyme, and as the second step adding IL). It was measured for *x*[LiTDI**·**y(4G)]:(1-*x*)BMImTDI system, where *y* = 1.0 and 4.5 and *x* = 0.050, 0.100, 0.300 and 0.500 at 20°C and 70°C.

| *x* | *η* / mPa s | | | |
| --- | --- | --- | --- | --- |
|  | *y*= 1.0  20°C | *y*= 4.5  20°C | *y*= 1.0  70°C | *y*= 4.5  70°C |
| 0.050 | 111.50 | 64.53 | 12.90 | 9.41 |
| 0.100 | 124.90 | 50.21 | 13.72 | 8.17 |
| 0.300 | 186.40 | 26.31 | 16.72 | 5.66 |
| 0.500 | 302.50 | 17.67 | 21.09 | 4.39 |
